# High bunch charge low-energy electron streak diffraction

**DOI:** 10.1063/4.0000246

**Published:** 2024-04-08

**Authors:** Chiwon Lee, Günther H. Kassier, R. J. Dwayne Miller

**Affiliations:** 1 Departments of Chemistry and Physics, University of Toronto, 80 St. George Street, Toronto, Ontario M5S 3H6, Canada; 2 Center for Free-Electron Laser Science CFEL, Deutsches Elektronen-Synchrotron DESY, Notkestrasse 85, 22607 Hamburg, Germany

## Abstract

For time-resolved diffraction studies of irreversible structural dynamics upon photoexcitation, there are constraints on the number of perturbation cycles due to thermal effects and accumulated strain, which impact the degree of crystal order and spatial resolution. This problem is exasperated for surface studies that are more prone to disordering and defect formation. Ultrafast electron diffraction studies of these systems, with the conventional stroboscopic pump–probe protocol, require repetitive measurements on well-prepared diffraction samples to acquire and average signals above background in the dynamic range of interest from few tens to hundreds of picoseconds. Here, we present ultrafast streaked low-energy electron diffraction (LEED) that demands, in principle, only a single excitation per nominal data acquisition timeframe. By exploiting the space–time correlation characteristics of the streaking method and high-charge 2 keV electron bunches in the transmission geometry, we demonstrate about one order of magnitude reduction in the accumulated number of the excitation cycles and total electron dose, and 48% decrease in the root mean square error of the model fit residual compared to the conventional time-scanning measurement. We believe that our results demonstrate a viable alternative method with higher sensitivity to that of nanotip-based ultrafast LEED studies relying on a few electrons per a single excitation, to access to all classes of structural dynamics to provide an atomic level view of surface processes.

## INTRODUCTION

The abrupt discontinuity that occurs in the formation of surfaces and interfaces imparts interesting new properties and functionalities for condensed phase systems. At the interface, atoms possess fewer nearest neighbors than those in their bulk counterpart, making them more prone to structural relaxation with lower barriers, leading to intrinsic catalytic properties. Surface phenomena caused by physicochemical interactions of such asymmetrically coordinated atoms deviate from those observed in bulk via three-dimensional intra-atomic interactions. These differences between bulk and surface phenomena have long been of interest in multidisciplinary fields^1–3^ and now attract mounting attention from light-induced surface processes (e.g., photocatalytic water-splitting), whose prime processes occur exclusively within the top few atomic layers on the ultrashort timescales associated with barrier crossings.^4–6^ Microscopic investigation of the discontinuities defined by surface boundary conditions demands a structural probe capable of resolving atomic details with the ultimate surface sensitivity. Low-energy electrons with kinetic energy on the order of 1 keV or less feature an Angstrom scale mean free path and exceptionally large elastic scattering cross section,^7–9^ unattainable from other high-energy atomic structural probes such as soft/hard X-ray or relativistic electrons. To date, low-energy electron diffraction (LEED) has been successfully applied to *in situ* surface characterizations, and recent efforts have expanded static LEED to the stroboscopic pump–probe measurement protocol, highlighting its potential in the study of non-equilibrium structural dynamics at surfaces.^10–16^

In the development of ultrafast LEED (ULEED), a key challenge lies in the spatiotemporal manipulation^14–16^ of the probe electrons to light up surface atomic motions in the relevant time scales with sufficient beam brightness. This difficulty arises from dispersive characteristics of low-energy electrons generated by a femtosecond pulsed laser photoinjection using photocathodes.^17,18^ Without spatial tailoring, the generated electron bunch diverges in the lateral direction, resulting in a blurred or broadened reflection at the detector plane and loss of spatial resolution. In high-energy ultrafast electron diffraction, the beam divergence is typically mitigated by a pre- or post-sample magnetic lens,^19^ yet hardly adaptable to LEED geometries with the larger angle elastic scattering^8^ and need for extremely small propagation distances from source to sample to minimize space charge broadening of the electron pulse and loss of time resolution. In static LEED, direct integration of an electrostatic lens into a DC cathode has been devised to enable the effective beam collimation. In ULEED, this is undesirable due to elongation of the bunch travel distance toward the diffraction target, thereby exacerbating the temporal broadening. A recent attempt to circumvent this ambivalent problem has been to utilize a point-source like nanometer-scale cathode, allowing to develop a millimeter or even sub-millimeter sized lens-coupled electron gun.^20,21^ This “miniaturized” electron gun significantly shortens the source-to-sample distance, achieving an instrument response of 1–2 ps without additional electron-optic techniques. However, the innate low-charge bunch (up to a few hundred electrons per bunch to avoid excessive space charge aberrations) generated from the nanometer sized emission area^22,23^ is applicable only to fully photo-reversible systems driven by large numbers of excitation cycles of the sample at high-repetition rates (10–100 kHz) to attain a reasonable diffraction quality with sufficient signal-to-noise ratio (SNR). Surface systems undergoing irreversible structural transitions upon photo-excitations, such as photocatalytic substrates,^24,25^ nanoconfined ionic liquids,^26,27^ or biological interface^28^ susceptible to cumulative heating or bleaching, are not accessible with the nanotip-based approach, which represents a major gap in the study of the main research domains of surface science.

Here, we present time-resolved low-energy electron streak diffraction by exploiting a compact ultrafast streak camera, readily implementable to the LEED geometry, that opens up the study of irreversible surface processes. Instead of the conventional time-scanning method in ULEED to obtain the dynamics, we implement the streaking technique^29–33^ with a high-charge low-energy electron bunch that incorporates time-varying structural information of a two-dimensional atomic layer within a temporal window up to tens of picoseconds long. With the well-characterized streak velocity produced by the transient electric field of the streak deflector, we retrieve this information from the measured intensities of streaked reflections for several timeframes, enabling to reconstruct a long-time structural dynamic that had been accessed by the nanotip-based ULEED with a high-repetition rate and a long data acquisition time.

## RESULTS AND DISCUSSION

**FIG. 1.**
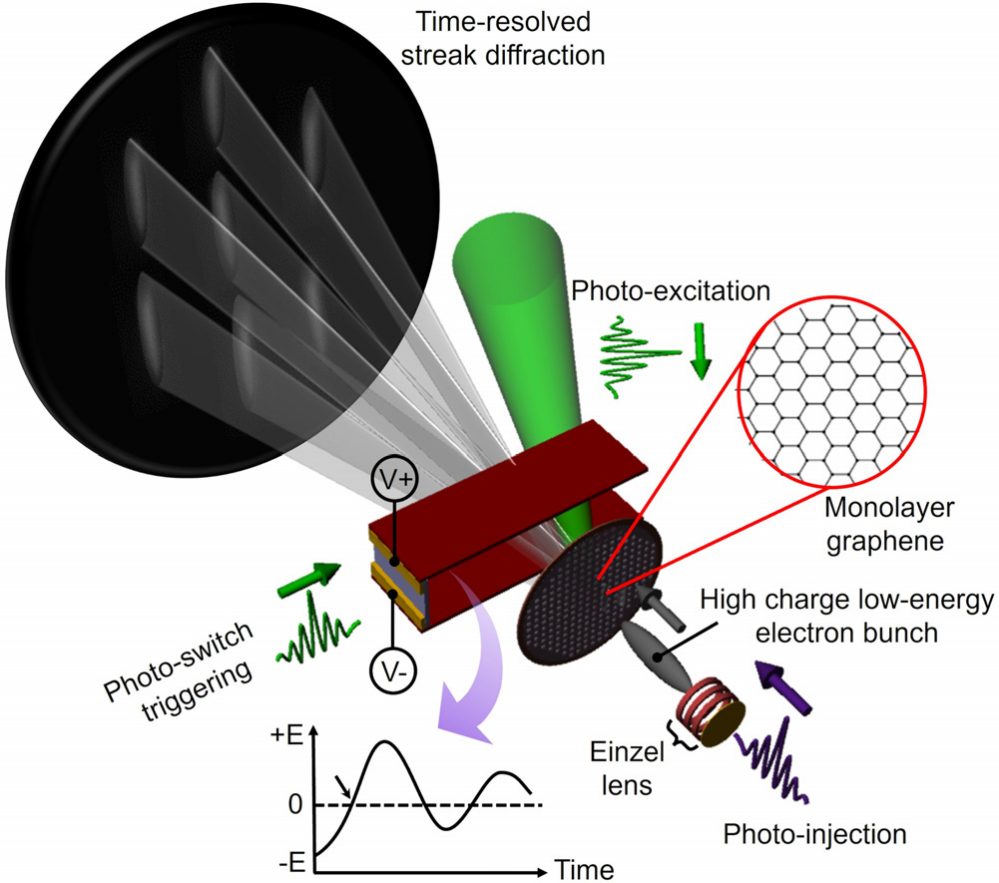
Schematic illustration of ultrafast low-energy electron streak diffraction. A damped oscillating electric field is generated inside the cavity between the upper and lower streak plates aligned in parallel, once the on-plate GaAs photo-switch is triggered by a femtosecond laser pulse for capacitive discharging from the positively and negatively charged plates. With careful control of the low-energy electron bunch entrance timing to the cavity with respect to the first field zero-crossing point (indicated as an arrow in the transient field plot), the probe electrons undergo the maximum sweep speed, resulting in the longest streak profile at the detector for a given charging voltage. The field at this region is approximated by a linear ramp. Additional synchronization with an optical excitation pulse allows for the investigation of atomic structural changes, such as inducing an increased RMS motion of the carbon atoms in freestanding graphene, as is done in the present study. The resultant time-resolved streak diffraction is obtained with a reduced number of excitation-probing cycles, showing an increased signal-to-noise-ratio.

Figure 1 illustrates our experimental scheme of the time-resolved ultrafast streaking of transmission-mode LEED. We generate a 2 keV electron bunch containing 10^5^ photoelectrons from a custom-made electron gun triggered by a pre-stretched 2.0 ps UV pulse running at 1 kHz, the master clock of the instrument. The beam current is measured and calibrated at the electron detector [microchannel plate (MCP)-phosphor screen assembly] used as an anode connected to a picoammeter, and the typical corresponding laser pulse energy is 23.5 nJ (Fig. S1).^39^ The high-charge bunch is, purposely, temporally broadened via the Coulomb repulsion during the drift space propagation within the source-to-sample distance (=∼5 mm), leading to the on-sample bunch duration of a few tens of picosecond. It has been shown that the inherent velocity dispersion for nonrelativistic electrons leads to exceptionally linear chirp and reproducible pulse profiles. The resulting stretched electron pulse duration defines, ideally in a single-shot streaking experiment, an observable time-window of the structural dynamics of interest. A home-built streak camera^34^ is placed ∼1 mm from the sample plane, designed to generate a damped harmonic oscillation electric field at a few GHz frequency inside the inter cavity gap (=∼1 mm) between two charged plates, one of which is connected to a semiconductor (GaAs) photo-switch. The switch is triggered by a second harmonic pulse split from the master signal, determining the onset time of the streak field. We find the first field zero-crossing at which the low-energy electron bunch transits the field symmetrically in time with zero net beam deflection by fine-controlling of the bunch entrance timing ( $t_{s}$) to the streak deflector. In this optimal timing-configuration, the electron bunch is expected to experience the maximum angular streak velocity $v_{\theta }$ for a given peak field strength and thereby produce streaking profiles that provide the maximum temporal resolution for a given time window. As a proof-of-principle, the ultrafast thermalization dynamics of a freestanding monolayer graphene is studied, where the relevant dynamics are characterized by the Bragg spot intensity modulations upon femtosecond laser excitation (i.e., second harmonic independently splits from the master source).

**FIG. 2.**
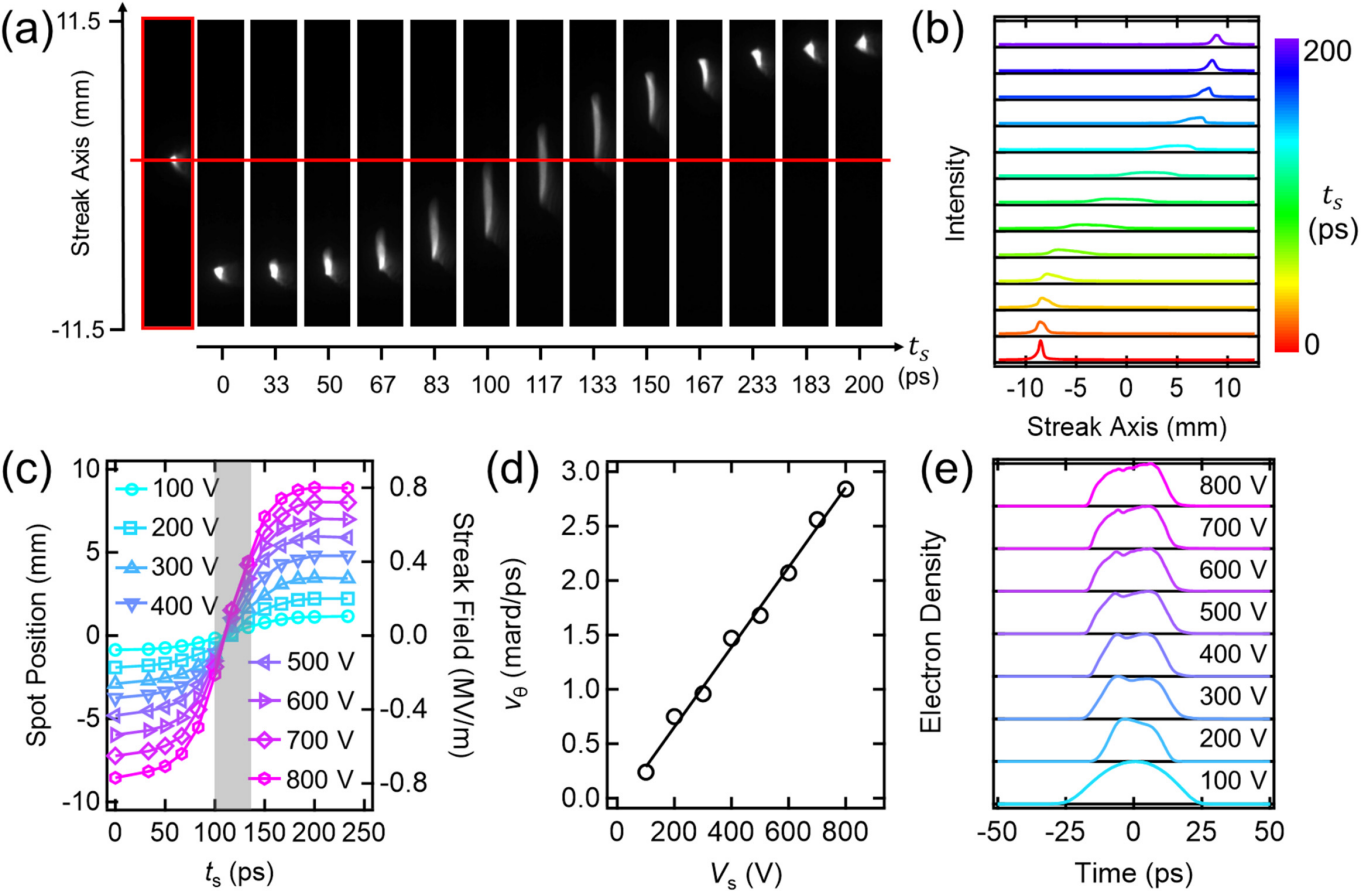
Streak camera characterization with direct beam. (a) Direct beam images taken as a function of $t_{s}$, for $V_{s}$ = 800 V. The red boxed image at the leftmost corner and the horizontal red line denote the unstreaked beam ( $V_{s}$ = 0 V) and its relative spot position with respect to the streak ones at the detector, respectively. The streaking direction is vertical and upward. (b) Intensity profile of the beam spots shown in (a), extracted after pixel binning in the normal direction of the streak-axis. The individual profile is plotted in the equally spaced y-axis without intensity normalization. Profiles for different $V_{s}$'*s* (= 100–700 V) are displayed in Fig. S2. (c) Summary of the spot position and temporally averaged streak field as a function of $t_{s}$ for different $V_{s}$'*s*. The angular streak velocity, $v_{\theta }$, is determined in the region approximated by a linear ramp-up (grey shaded). (d) $v_{\theta }$ vs $V_{s}$. (e) Temporal electron density profile for different $V_{s}$'*s*, extracted by deconvoluting the maximum streak profile with the unstreaked one of the direct beam images under the Tikhonov regularization framework.

To characterize our streak camera performance, we first record direct beam images by varying $t_{s}$, from which an integrated intensity profile is extracted along the perpendicular direction of the streak axis. As shown in Figs. 2(a) and 2(b), depending on $t_{s}$, beam deflection and shape modulation are observed with respect to the unstreaked one, resulting in the intensity profile changes of the streak spots. We systematically investigate this streaking effect on the beam spot position for different streak plate voltages, $V_{s}$, in the range of 100–800 V. In Fig. 2(c), the measured spot position is plotted as a function of $t_{s}$ for the respective $V_{s}$, which retrieves a temporal average of the electric field integrated over the bunch transit time through the streak deflector plates. From this plot, we obtain the cavity resonance frequency to be ∼2.5 GHz and determine $v_{\theta }$ by fitting data points in proximity to the zero-crossing region ( $t_{s}$ = 100–130 ps). As summarized in Fig. 2(d), $v_{\theta }$ is increased with the increase in $V_{s}$, ascribed by the proportional relation between the beam deflection and the applied field strength for the given bunch transit time.

For the direct beam measurement, deconvoluting a streak spot intensity profile with the unstreaked one yields a temporal profile of the electron bunch. We take the net-zero deflected streak profile (corresponding to $t_{s}$ = 117 ps) from the $V_{s}$ datasets and adapt the Tikhonov regularization framework to solve the ill-posed deconvolution problem, which was implemented for the earlier work with the 30 keV direct electron beam.^34^ To avoid overfitting, the regularization parameter is chosen such that a root mean square error (RMSE) between the regularized streak profile and the unregularized one is within our measurement uncertainty (∼2%) taken from the variance of the streak profile intensities calculated from multiple streak images. The extracted electron density profiles are displayed in Fig. 2(e), from which the full-width-half-maximum (FWHM) bunch duration is determined. The streak camera temporal resolution for the respective $V_{s}$ case is deduced from the finite temporal size of the unstreaked spot within which time-dependent information is unresolvable (Table I). We select the $V_{s}$ = 300 V case and take the bunch duration as the dynamic range of a single streak in the time-resolved study (see later).

**TABLE I.** FWHM bunch duration and measurement resolution determined from the temporal profiles [Fig. 2(e)] as a function of $V_{s}$.

$V_{s}$ (V)	100	200	300	400	500	600	700	800
Bunch duration (ps)	31.2	19.7	25.3	23.2	26.1	25.8	26.4	25.8
Resolution (ps)	20.1	6.6	5.1	3.4	2.9	2.4	1.9	1.7

**FIG. 3.**
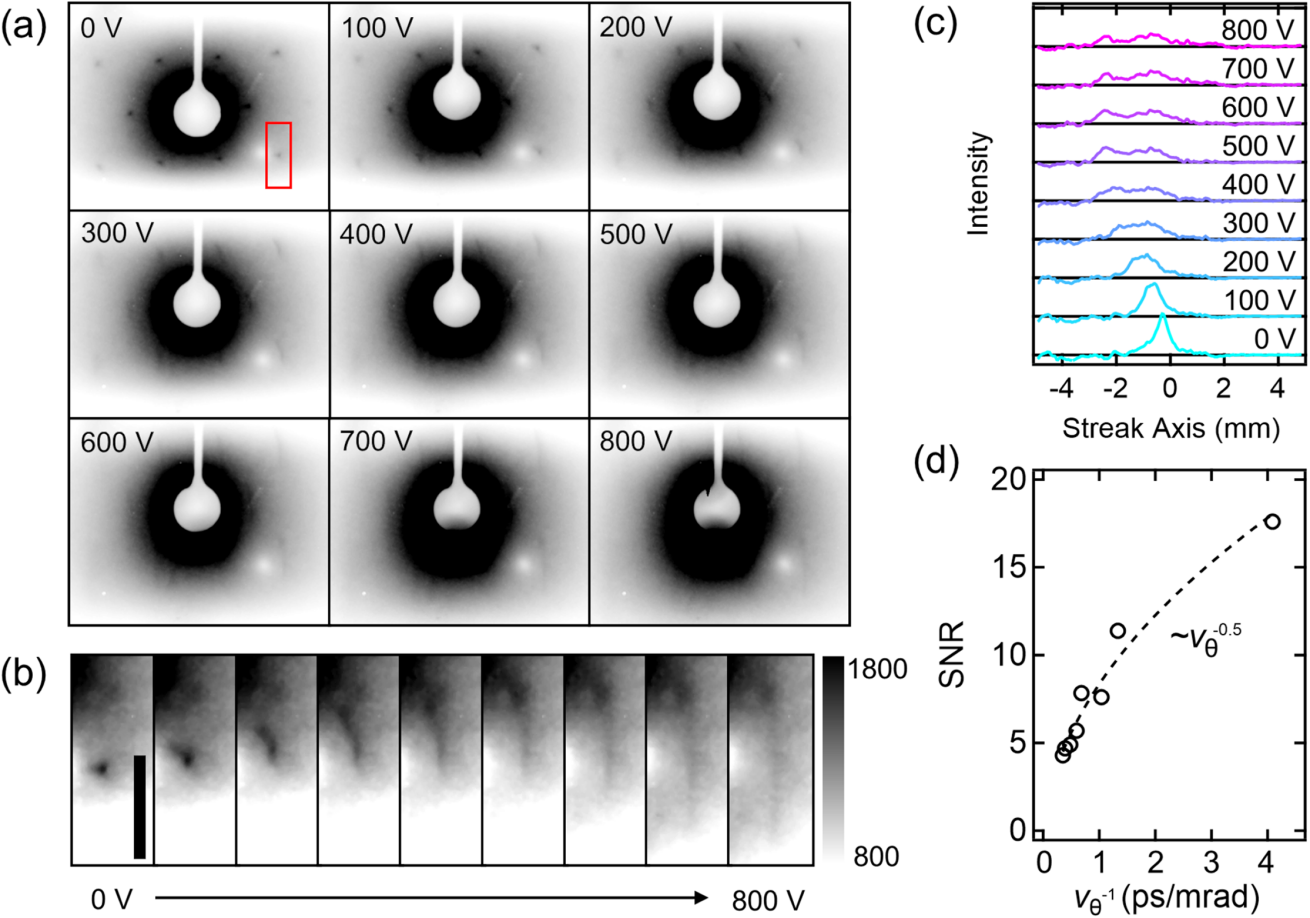
Streak low-energy electron diffraction of monolayer graphene. (a) Streak diffraction images for different $V_{s}$'*s*. (b) Zoom-in image of the ROI indicated by the red box in (a), highlighting the reflection spot evolution as function of $V_{s}$. Scale bar: 3 mm. (c) Background subtracted intensity profile of the beam spots shown in (b). The individual profile is displayed in the equally spaced y-axis without intensity normalization. (d) SNR of the intensity profile as a function of the inverse of the angular streak velocity, $v_{\theta }^{-1}$. The dotted line indicates the power regression fit to the extracted values, showing an inverse of square root dependence of SNR on $v_{\theta }$.

Next, we place a freestanding graphene monolayer suspended on a copper mesh grid in front of the streak camera and repeat the measurement at the net-zero deflection condition for different $V_{s}$, yet with a longer image acquisition time and larger microchannel plate (MCP) gain for the appropriate visualization of the elastically scattered electrons (i.e., Bragg spots) at the detector. Detailed experimental settings are described in the supplementary material. In our experimental configuration, we observe that the second order reflections are partially apertured by the upper and lower streak plates, while the sixfold symmetric first order ones are all visible at $V_{s}$ = 0 V. As shown in Figs. 3(a) and 3(b), with the increase in $V_{s}$, the shape of the Bragg spots is elongated, and, concomitantly, the spots become less visible due to the overlap with the inelastic scattering background. This background smearing is more pronounced for small angle scattering spots that are closer to the direct beam center.

We take a region-of-interest (ROI) for one of the second order spots [as indicated by the red box of the $V_{s}$ = 0 V panel in Fig. 3(a) for the intensity profile analysis]. Pixel integration of the ROI along the perpendicular direction of the streak-axis yields an intensity profile composed of both (1) the signal-of-interest and (2) an inhomogeneous baseline caused by the inelastic background that is also streaked. To remove the arbitrary baseline, we first preprocess the raw profile by subtracting the average of the two profiles, respectively, pixel-integrated on the right- and left-side region adjacent to the streak spot. Subsequently, we apply the iterative discrete wavelet transform (DWT), a multiresolution decomposition technique allowing for the frequency analysis with different spatial resolution.^35,36^ A converged baseline is obtained after multiple times of iteration of DWT, again subtracted from the preprocessed data. The resultant background subtracted streak diffraction profile is displayed for different $V_{s}$ s in Fig. 3(c), capturing the effect of streaking on the elastically scattered electrons; as similar with the direct beam case, with the increase in $V_{s}$, the spot profile is elongated, resulting in the decrease in the peak intensity, $I_{\text{peak}}$. We also calculate the standard deviation of intensities, $\sigma_{I}$, of a part of the respective profile, in which no apparent signals are present, and evaluate SNR defined by $\frac{I_{\text{peak}}}{\sigma_{I}}$. The result is plotted as a function of the inverse of the angular streak velocity, $v_{\theta }^{-1}$, extracted from the direct beam measurement at the corresponding $V_{s}$ condition, as shown in Fig. 3(d). We find a square root dependence of SNR on $v_{\theta }^{-1}$, following Poisson statics, given the linearly inverse relation between the bunch charge density and $v_{\theta }$. This approach gives a more adequate noise model relevant to the time-dependent intensity reconstruction, for example, for the image analysis of powder or overlapped streak diffraction, than a Gaussian random noise assumption posed by our earlier work.^32^

**FIG. 4.**
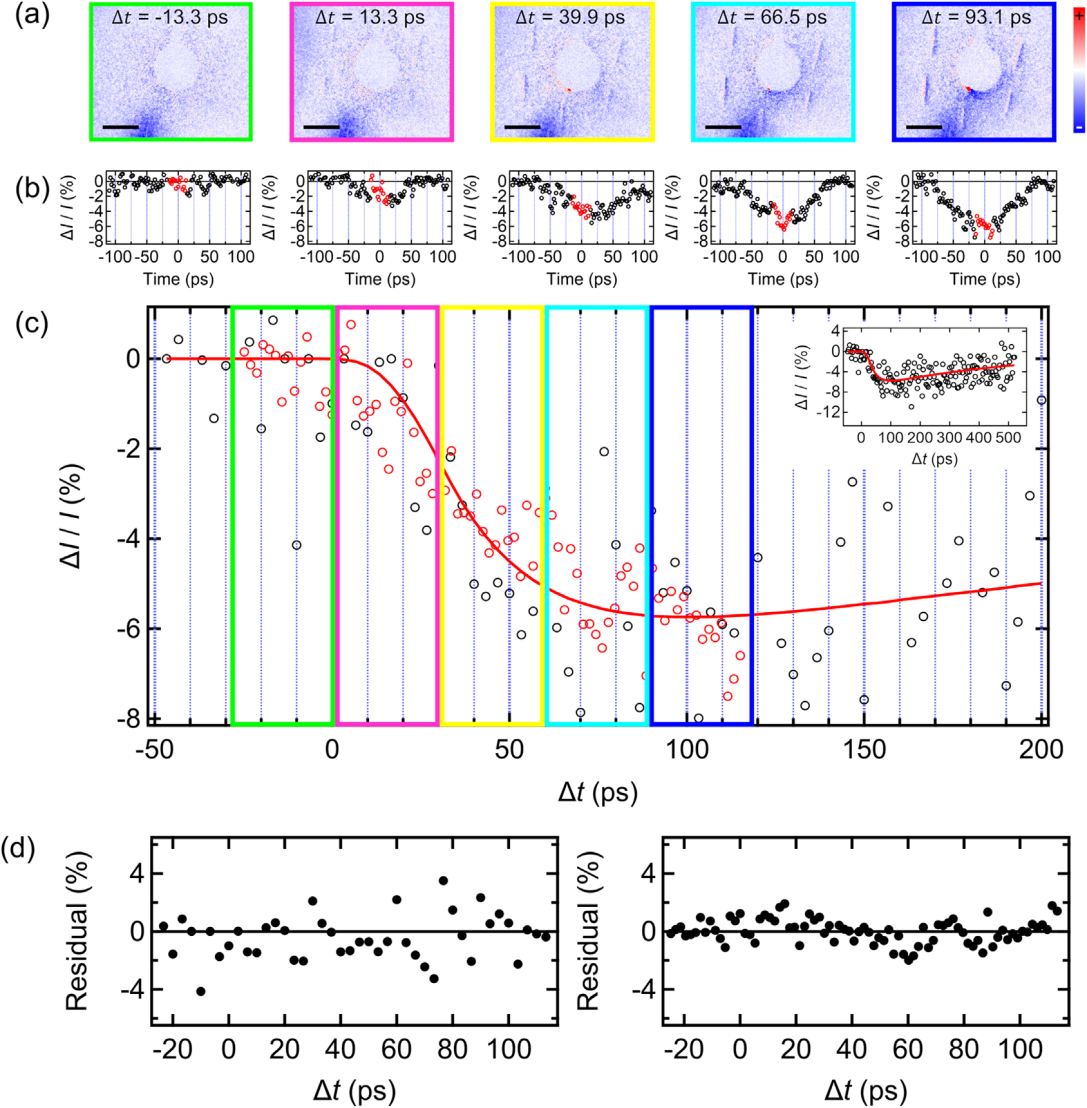
Time-resolved streak low-energy electron diffraction. (a) Intensity difference $\left[\Delta I\;(=I_{\Delta t>0}-I_{\Delta t<0}\right)$] map of the streak diffraction of graphene upon laser excitation for five different $\Delta_{t}$'*s* (=−13.3, 13.3, 39.9, 66.5, and 93.1 ps). A highlighted region for the first order reflection streaks is displayed in Fig. S6. (b) Temporal profile of the relative intensity difference ( $\Delta I/I_{\Delta t<0}$) of the first order streak reflections. The red circles of the respective profile indicate the data segment sampled to reconstruct the long-range intensity time-trace shown in (c). The time-axis of the plots is calibrated using the measured $v_{\theta }$. (c) Comparison of the intensity time-trace obtained from the time-resolved ultrafast low-energy electron streak diffraction (red circle) and the conventional time-scanning pump–probe ULEED (black circle) independently measured. The red spline is the model fit extracted from the scanning ULEED as shown in the inset (upper right-side corner). Note that the border color (green, magenta, yellow, sky blue, and dark blue) of the respective box is set to show the one-to-one correspondence between the respective temporal window of the reconstructed time-trace and the intensity difference map where the data points are extracted, as indicated in the identical color in (a). (d) Fit residual plot for the scanning ULEED (left panel) and the streak diffraction (right panel).

We then, in the streaking-mode, photo-excite the graphene layer by ultrashort laser pulses with the time delay ( Δt) between the optical pulse and the electron bunch at the sample plane. We acquire five streak images at a constant Δt interval (= 26.6 ps) with and without the excitation and obtain the intensity difference [ $\Delta I\;(=I_{\Delta t>0}-I_{\Delta t<0})$] map [Fig. 4(a)]. From the respective map, intensity profiles of the first order visible streaks are extracted (along the streak direction) and averaged, resulting in the temporal profile of the relative intensity difference ( $\Delta I/I_{\Delta t<0}$), as shown in Fig. 4(b). From this profile, we take the data points in the dynamic range (=26.6 ps) of interest whose center point matches with the profile center (i.e., time = 0 ps) and stitch the segment of the respective profile next to each other in consecutive order, leading to a reconstructed profile that covers ∼140 ps of the dynamic range in total [Fig. 4(c)]. Independent from the streaking measurement, we also carry out a conventional stroboscopic time-scanning diffraction with the same excitation condition yet at a finer Δt interval (=3.3 ps) for a longer time window (=570 ps). The measured scanning data are fitted into a double-exponential model that captures both the rapid thermal heating and a subsequent slow anharmonic phonon mode relaxation of the graphene lattice upon photoexcitation,^13^ which is convolved with the measured temporal electron density profile in Fig. 2(e) (see supplementary material for more details). The resultant model fit is compared with the scanning and streaking data individually, giving rise to a residual fit plot, as shown in Fig. 4(d).

Table II presents statistics on parameters of the respective measurement. Given that the same dynamic range of interest is covered by 5 and 42 timeframes for the streaking and scanning measurement, respectively, with the same bunch charge for both measurements, we find an 88.1% reduction in the total number of electrons accumulated for the entire course of the data acquisition in the streaking over the scanning for comparable SNR. Moreover, even with the reduced number of the accumulated probe electrons, the reconstructed dynamics from the 5 individual temporal profiles shows 48.0% smaller RMSE from the fit model, calculated from the residual plot, compared to that of the scanning measurement. This feat is directly attributed to the space–time correlation characteristics of the streaking measurement and the low timing jitter of our streak camera. In addition, we note that the total number of the sample excitation cycles and the elapsed time during the streaking measurement are reduced proportionally by the decrease in the number of the acquisition timeframes needed for comparable SNR between the respective methods. This implies that streak diffraction with high bunch charge electrons could significantly reduce both measures compared to the nanotip-based ULEED that demands several orders of magnitude more sample excitation cycles due to the low-charge density characteristics of the electron bunch, even considering the mitigated effect by the larger transverse coherence. Finally, the temporal resolution of the streak diffraction is evaluated at 5.5 ps, which is dominated by the streak camera temporal resolution (=5.1 ps) at the set $V_{s}$ condition (=300 V) of the present measurement; however, we note that, with the larger gain in streak velocity at higher $V_{s}$ (as demonstrated in the $V_{s}$ = 800 V case), a further reduction down to 1–2 ps is possible, which could even extend to the sub-picosecond regime with increased bunch brightness. This time resolution is sufficient to follow most surface processes of interest.

**TABLE II.** Summary of the measurement parameters.

	Scanning	Streaking
Dynamic window of interest (ps)	140	140
Total number of timeframes	42	5
Number of electrons per bunch	$1\times 10^{5}$	$1\times 10^{5}$
Number of electrons per timeframe	8 $\times 10^{8}$	8 $\times 10^{8}$
Total number of accumulated electrons	$3.36\times 10^{10}$	$4\times 10^{9}$
Number of excitation cycles per timeframe	8 $\times 10^{3}$	8 $\times 10^{3}$
Total number of accumulated excitation cycles	$3.36\times 10^{5}$	$4\times 10^{4}$
RMS error (%)	1.584	0.829
Elapsed time of total image acquisition (s)	1528.8	182.0
Temporal resolution (ps)	25.3	5.5

The presented results suggest several routes toward an ideal single-shot measurement (i.e., single excitation per single acquisition timeframe) in time-resolved LEED. First, recent advancements in photocathode technology relying on near-threshold photoemission from single-crystalline materials at cryogenically cooled temperature have shown a single digit meV mean-transverse-energy of photoelectrons;^37,38^ the resultant bunch emittance is expected to substantially decrease over that of the typical photocathode made of a polycrystalline metal film, operating at room temperature. Second, increasing the photoinjection trigger pulse duration and employing a more optimal temporal pulse shape would allow higher charge bunches with an on-target bunch duration comparable to the present bunch parameter. For example, replacing the 2.0 ps long Gaussian pulse used in the present setup with a flat-top one would lower the peak power density of the injection pulse, which is then temporally stretched up to tens of picosecond; this manipulation would allow the injection using more photons and resulting photoemitted electrons for a longer emission time window while mitigating space charge induced growth of the bunch duration and avoiding undesired multiphoton emission and laser heating effects at the photocathode.^33^ Implementing these two ideas into our current setup should give a significant increase in bunch brightness in total, indicating the feasibility of the single-shot regime for transmission-mode LEED. Third, for the back-scattering geometry that has less than 20% scattering intensity compared with that of the transmission-mode one,^8^ raster scanning and signal averaging over multiple spots of the diffraction target are desirable. Given the excitation beam spot size (=∼300 *μ*m) of our setup, the total area of a standard TEM grid (∼3 mm) can provide nearly 75 fresh spots. These spatial profiles make alignment relatively simple, but smaller beam diameters are easy to implement to increase sampling as needed. The present beam geometry is sufficient to collect visible signals at several timeframes. MCP gating to reject detection noise in between the signal arrival periods could further improve SNR in streaked images.

## SUMMARY

In summary, we have presented ultrafast streaking of the LEED patterns by adapting a compact streak camera and demonstrated the substantial improvement of SNR over the conventional time-scanning method to investigate atomic structural changes of monolayer graphene upon ultrashort laser excitation. This alternative approach over nanotip-based ULEED has showed the significant reduction in the sample excitation cycle, electron dose, recording time frame, and total measurement time in capturing more than one hundred picosecond long dynamics, thus allowing us to extract structural information from atomic boundaries whose ground state is not fully replenished upon photoexcitation. This approach enables fully resolving the temporal dynamics and meeting SNR requirements for the desired atomic spatial resolution within typical sample constraints. Future work with the outlined ideas to enhance the bunch brightness of low-energy photoelectrons holds promise to fully open up access to the generally irreversible dynamics directing surface and interfacial phenomena.

## Data Availability

The data that support the findings of this study are available from the corresponding author upon reasonable request.
